# LHPP suppresses proliferation, migration, and invasion and promotes apoptosis in pancreatic cancer

**DOI:** 10.1042/BSR20194142

**Published:** 2020-03-27

**Authors:** Fahong Wu, Yanling Chen, Jinhai Zhu

**Affiliations:** Department of Hepatobiliary Surgery and Fujian Institute of Hepatobiliary Surgery, Fujian Medical University Union Hospital, Fujian Medical University, Fuzhou, China

**Keywords:** apoptosis, invasion, LHPP, migration, pancreatic cancer, proliferation, PTEN/AKT

## Abstract

Pancreatic cancer (PaCa) is a common malignant tumor of the digestive system with poor prognosis and no ideal treatment for inoperable patients, which is partly due to delayed diagnoses. It is recently reported that the protein histidine phosphatase LHPP is a tumor suppressor in hepatocellular carcinoma, cervical cancer, and bladder cancer. So far, there is no study on the expression level of LHPP in PaCa, and its mechanism of action on tumors is unclear. In this experiment, LHPP expression was lower in cancer tissues than that in normal pancreatic tissue, and clinicopathological results showed that LHPP expression was correlated with the degree of differentiation and lymphatic metastasis of pancreatic carcinoma. The biological characteristics of LHPP in PaCa cells were examined by the cell counting kit-8 assay, transwell assay, and monoclonal formation test. The inhibitory mechanism of LHPP in PaCa cells was determined using Western blotting and flow cytometry. The results showed that LHPP restrained PaCa cell proliferation, migration, and invasion. Increased LHPP expression promoted the apoptosis of PaCa cells through higher activation of cleaved-PARP and cleaved-Casp3 and lower activation of cIAP1. Importantly, the increase in LHPP enhanced PTEN expression and decreased the phosphorylated AKT level. Moreover, LHPP-induced apoptosis was diminished by SC79 (AKT activator) in PaCa cells. In conclusion, LHPP blocks proliferation, migration, and invasion and enhances apoptosis in PaCa cells through the PTEN/AKT signaling pathway.

## Introduction

Pancreatic cancer (PaCa) is one of the most invasive malignant tumors. Presently, radical resection is the first choice for patients with pancreatic cancer and is considered the only method for achieving long-term survival in patients. However, the resection rate is only 15–20% and the 5-year survival rate is less than 7%, which is partly due to delayed diagnoses since more than half of patients are diagnosed at advanced stages [1,2]. At present, although the surgical technique has been greatly improved, the survival rate has not changed significantly. It is reported that 70% of patients with PaCa have metastasis in resected specimens, and up to 80% of patients eventually experience local recurrence [3,4]. Therefore, it is becoming increasingly urgent to identify an effective treatment and diagnostic method for PaCa. We must explore new treatments and the mechanism of PaCa tumor development to provide a new strategy for its treatment.

Phospholysine phosphohistidine inorganic pyrophosphate phosphatase (LHPP) was first detected in swine brain tissue. [5,6]. Protein phosphorylation plays an important role in epigenetics, which is considered to be a powerful means of regulating carcinogenic activity [7,8]. Recently, the anti-tumor effect of LHPP has been further demonstrated: LHPP is considered as a tumor suppressor gene of hepatocellular carcinoma by the PI3K/AKT signaling pathway, and its low expression leads to a decrease in patient survival rate [9]. In cervical cancer, it is also found that LHPP can promote apoptosis by increasing the expression of cleaved-PARP and cleaved-Casp3 protein [10]. As a new tumor suppressor, the anti-tumor effect of LHPP in melanoma and bladder cancer has been further confirmed [11,12]. However, the anti-tumor effect of LHPP on PaCa has not been verified.

In the present study, we aimed to examine the relationship between LHPP and PaCa development. Our findings suggested that LHPP expression was lower in cancer tissues than that in normal pancreatic tissue, and its expression was also related to the degree of tumor cell differentiation and lymphocyte metastasis. Functional studies have shown that LHPP overexpression reduced the proliferation, migration, and invasion and enhanced apoptosis of PaCa cells, and the opposite result was observed with LHPP knockout. We also found that LHPP could inhibit the AKT pathway in PaCa cells. Thus, our results showed that LHPP inhibited PaCa development and served as a potential tumor target gene, which will provide a new strategy for the treatment of PaCa

## Material and methods

### Tissue samples and cell lines

In total, 36 PaCa tissues and matched normal tissues were obtained from Union Hospital of Fujian Medical University, and patients provided informed consent before operation. All PaCa tissues were diagnosed by clinical pathology. Human PaCa cell lines (BxPC-3 and AsPC-1) and pancreatic ductal cell line HPDE6–C7 were acquired from the Institute of Hepatobiliary Surgery of Union Hospital and were cultured separately in DMEM with 12% FBS (Gibco, Grand Island, NY, U.S.A.).

### Immunohistochemistry

Immunohistochemistry was performed as previously described [13]. Primary antibodies for LHPP (1:200, Invitrogen, Carlsbad, CA, U.S.A.) were incubated with tissue sections overnight at 4°C, and five random fields per section were captured at the same exposure value under an optical microscope (Olympus, Tokyo, Japan). LHPP expression was evaluated by the following methods: Mean Optical Density (MOD) = Integral Optical Density (IOD)/Positive Area. Each field was scored independently by two pathologists.

### Quantitative real-time polymerase chain reaction

Total RNA was extracted from cells with TransZol UP reagent (Transgene, Strasbourg, France) and reverse transcribed with the Revert Aid First Strand cDNA Synthesis Kit (Transgene). The following q-PCR primer sequences were used: LHPP forward: 5-CAAACTGTGTGGTAATTGCAGA-3, reverse: 5-CCAGAGGTCTCCTTGTAGTAAC-3; snail forward: 5-CCTTCGTCCTTCTCCTCTACTT-3, reverse: 5-GCTTCGGATGTGCATCTTGA-3; laminin-5 forward: 5-CCATGAATTTCTCCTACTCGCC-3, reverse: 5-CTCCGATGCTGATATCCTTGAT-3; L1CAM forward: 5-GCTGGTTCATCGGCTTTGTG-3, reverse: 5-CTGTACTCGCCGAAGGTCTC-3; N-cadherin forward: 5-CGATAAGGATCAACCCCATACA-3, reverse: 5-TTCAAAGTCGATTGGTTTGACC-3; vimentin forward: 5-AGTCCACTGAGTACCGGAGAC-3, reverse: 5-CATTTCACGCATCTGGCGTTC-3; E-cadherin forward: 5-AGTCACTGACACCAACGATAAT-3, reverse: 5-ATCGTTGTTCACTGGATTTGTG-3;GAPDH forward: 5-CACCCACTCCTCCACCTTTGA-3, reverse: 5-TCTCTCTTCCTCTTGTGCTCTTGC-3. PCR was performed with Fast Start Universal SYBR Green Master Mix (Roche, Basel, Switzerland), and the fluorescence was measured using an ABI 7500 Real Time System (Applied Biosystems, Life Technologies, Foster City, CA, U.S.A.) by following the manufacturer’s instructions. Data were analyzed using the 2^−ΔΔCt^ method, and GAPDH was regarded as an internal control.

### Cell transfection and treatment

The synthesized overexpression vector of LHPP (LHPP) and negative control (Vector) were annealed and ligated into the pGLVH1/RFP/Puro plasmid, and the overexpression vector of the human LHPP sequence (NM_001167880.2) was bought from GenePharma (Shanghai, China). For LHPP knockdown (shLHPP), shRNA against LHPP was designed by Hanbio (Shanghai, China), and the lentivirus without the transgene was produced in the same manner and used as a negative control (NC). The shLHPP sequence was 5′-CCGCTCAGAATTTGATCAGAT-3′. Transfection of the LHPP and shLHPP groups was performed in OptiMEM medium (Life Technologies) using Polybrene (GenePharma) when cells reached 30–50% confluence, in accordance with the manufacturer’s protocols. After the lentivirus vector was transfected into the BxPC-3 and AsPC-1 cells, the stable cell lines were obtained with puromycin treatment for 2 weeks. For the AKT activator, SC79 was bought from MedChemExpress. The LHPP group cells were pretreated with SC79 in 8 μg/ml.

### Cell proliferation assay

PaCa cell viability was determined by the Cell Counting Kit-8 (CCK-8) assay according to the manufacturer’s instructions (Dojindo Laboratories, Kumamoto, Japan). In brief, 2 × 10^3^ cells/well were inoculated into 96-well plates, and 10 μl CCK-8 and 90 μl medium reagent were added to each well, followed by incubation for 1.5 h at 37°C. The results were determined by a plate reader at 450 and 600 nm at intervals of 24, 48, 72, and 96 h (Bio-Rad, Hercules, CA, U.S.A.).

For colony formation assay, 1 × 10^3^ cells/well were inoculated into 6-well plates and cultured for 10 days. The plates were fixed with methanol and then dyed with Crystal Violet solution for 30 min. Finally, the plates were photographed.

### Cell migration assay and cell invasion assay

The cell migration and invasion ability was analyzed using a 24-well transwell chamber with 8 μm polycarbonate membranes (Millipore, Washington, DC, U.S.A.). For the cell invasion assay, the chamber above the membrane was coated with matrigel (20 μg/well) (BD Biosciences, Lake Franklin, NJ, U.S.A.) and then air-dried for 2 h at 37°C. Cells were inoculated at 2 × 10^5^ cells/well in the upper chambers with serum-free DMEM, and the lower chamber was filled with 12% FBS. After incubating for 48 h, cells that passed through the membranes were fixed with paraformaldehyde and dyed with Crystal Violet. The cells in the lower chambers were counted in five random fields. Each condition was repeated in triplicate.

### Western blotting

Cells transfected with lentiviral vectors were lysed with lysis buffer at 4°C for 30 min. The concentration of cells was measured by the BCA Protein Assay Kit (Beyotime Institute of Biotechnology, Shanghai, China). Proteins were separated by SDS-PAGE and transferred to polyvinylidene fluoride membranes, which were then sealed with non-fat milk in tris-buffered saline with Tween for 3 h. The appropriate diluted primary antibodies, including anti-human LHPP (1:500; Invitrogen Carlsbad, CA, U.S.A.), anti-human cIAP1 (1:1000; Cell Signaling Technology, Danvers, MA, U.S.A.), cleaved anti-human Caspase-3 (1:500; Abcam, Cambridge, U.K.), cleaved anti-human PARP (1:1000; Cell Signaling Technology), anti-human p53 (1:1000; Cell Signaling Technology), anti-human AKT-pS473 (1:3000; Proteintech, Chicago, IL, U.S.A.), anti-human AKT (1:1000; Cell Signaling Technology), anti-human PTEN (1:1000; Cell Signaling Technology), and anti-human GAPDH (1:2000; Servicebio, Wuhan, China) were then incubated with the membranes overnight at 4°C. The second antibody was diluted with TBST and incubated with the membranes for 1 h at room temperature. Immunoreactivity was determined using a chemiluminescence western blot immunodetection kit (Invitrogen) according to the manufacturer’s instructions. The amounts of each protein were semi-quantified, and GAPDH was regarded as an internal control.

### Cell apoptosis assay

The apoptosis rate of PaCa cells was measured by flow cytometry. Cells with good viability were placed in a six-well plate. Then, cells were dissociated, washed, and bound to PE-A (Phycoerythrin-area) and PerCP-A (Chlorophyllin-area) according to the manufacturer’s instructions. The fluorescence intensity was determined by a BD Accuri C6 flow cytometer (BD Biosciences, San Jose, CA, U.S.A.).

### Statistical analysis

The data were expressed as means ± SD. GraphPad Prism V7.0 was used to produce column graphs, and FlowJo v10 was used to analyze the percentages of apoptotic cells and produce dot plots. Data were processed by SPSS software (version 22.0). Fisher’s exact test was applied to the quantitative data, the *t*-test was used to compare mean values between the two groups, and one-way analysis of variance was used to compare the mean values between multiple groups. *P* < 0.05 was considered statistically significant.

## Result

### Low LHPP expression in pancreatic carcinoma tissues and cell lines

**Figure 1:**
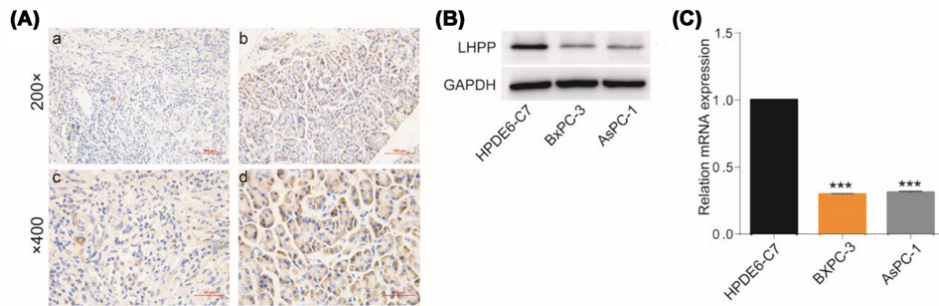
Low LHPP expression in pancreatic carcinoma tissues and cell lines (**A**) (**a** and **c**) LHPP protein expression was low in PaCa tissues. (**b** and **d**) LHPP protein expression in normal pancreatic tissues was increased. (**B** and **C**) The protein and mRNA expression levels in BxPC-3 and AsPC-1 cells were significantly lower than those in the HPDE6-C7 cells (***P <0.001).

We performed immunohistochemical analyses of PaCa tissues and matched normal tumor tissues from 36 patients to investigate the LHPP expression levels. As shown in Figure 1A, LHPP expression was lower in cancer tissues than that in normal pancreatic tissue. Upon further immunohistochemical analysis, the results showed that PaCa tissues were poorly dyed with an MOD of 0.0138 ± 0.0697, and the normal pancreatic tissues were well dyed with an MOD of 0.0225 ± 0.0105 (Table 1). Additionally, the protein and mRNA expression levels in BxPC-3 and AsPC-1 cells were significantly lower than those in HPDE6-C7 cells (Figure 1B,C). Collectively, these results revealed that LHPP was significantly down-regulated in PaCa tissues and cells.

**Table 1:** The expression of LHPP measured by MOD in PaCa tissues and matched non-tumor tissues

	*N*	LHPP expression MOD (mean ± S.D.)	*T*-value	*P*
Non-tumor tissues	36	0.0225 ± 0.0105	4.392	<0.001
PaCa tissues	36	0.0138 ± 0.0697		

### Relationship between LHPP expression and clinicopathological characteristics in pancreatic carcinoma

We further analyzed LHPP expression and the clinicopathological features of PaCa (Table 2). It was found that the degree of differentiation of pancreatic carcinoma was negatively correlated with LHPP expression (*P* = 0.018), the differentiation ability of the group with low expression of LHPP was significantly enhanced, while that of the group with high expression of LHPP was significantly weakened. LHPP expression in pancreatic carcinoma with lymph node metastasis was significantly lower than in that without lymph node metastasis (*P* = 0.010). It was suggested that the decrease in LHPP in PaCa may be related to the increased invasion of cancer cells to lymphoid tissue. We examined the relationship between TNM stage and LHPP expression in 36 patients who underwent surgery after 2016, although statistical correlation was not evident between them (*P* = 0.305). There were no differences in age, gender, tumor size, tumor location, nerve metastasis, and vascular invasion between the groups (*P* > 0.05).

**Table 2:** Correlation of clinicopathological features of LHPP in 36 patients

Characteristics	All patients	LHPP expression		*P* value
		Low	High	
No.	36	28	8	
Age (years)				0.244
<60	20	17	3	
≥60	16	11	5	
Gender				0.786
Male	21	16	5	
Female	15	12	3	
Tumor size (cm)				0.980
≤2	6	5	1	
2–4	15	11	4	
>4	15	12	3	
Tumor location				0.927
Head	22	17	5	
Body/Tail	14	11	3	
Diffentiation				0.018
Well-moderate	22	20	2	
Poor	14	8	6	
Lymph node metastasis				
Present	19	18	1	0.010
Absent	17	10	7	
Nerve metastasis				0.532
Yes	19	14	5	
No	17	14	3	
Vacular invasion				0.486
Yes	10	7	3	
No	26	21	5	
TNM stage				0.305
I/II	31	25	6	
III/IV	5	3	2	

### LHPP inhibits proliferation, migration, and invasion of pancreatic cancer cells

**Figure 2:**
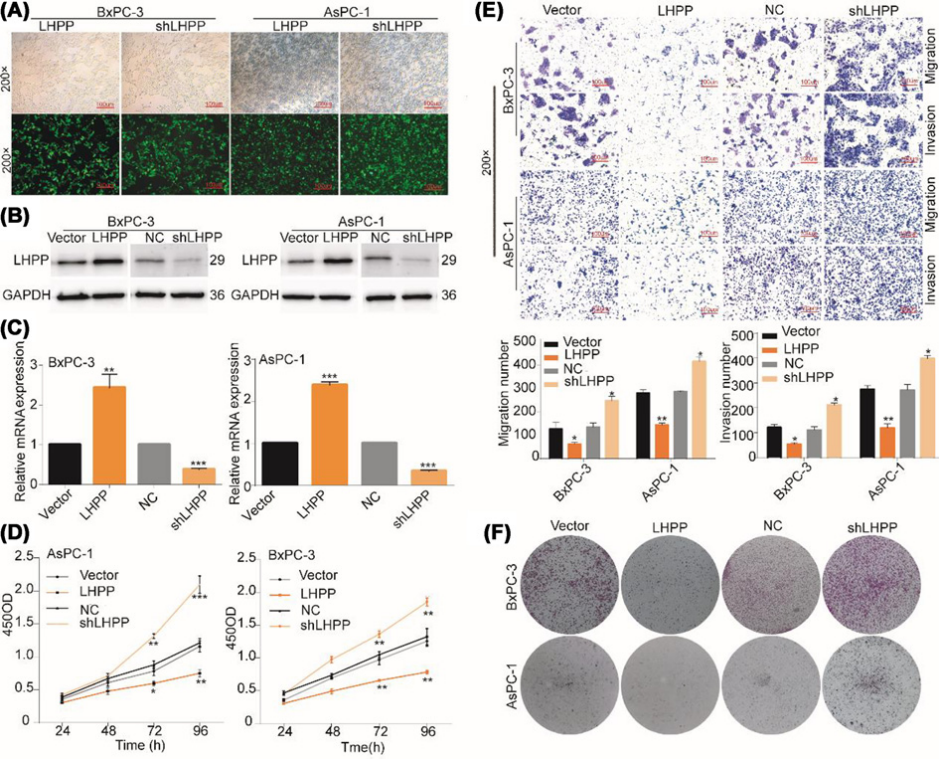
LHPP suppressed proliferation, migration, and invasion in pancreatic cancer cells (**A**) The designated stably lentivirus-transfected cells, including BxPC-3 and AsPC-1, were established as LHPP and shLHPP, and after 72 h of lentivirus transfection, the transfection efficiency of the two cell lines was more than 90% (*P* <0.001). (**B**) To the BxPC-3 cells, the LHPP protein levels in the overexpression groups (1.2109 ± 0.0118) were significantly higher than those in the control groups (0.6238 ± 0.0332), the LHPP protein levels in the knockdown groups (0.2774 ± 0.0170) were significantly lower than those in the control groups (0.5736 ± 0.0305) to the AsPC-1 cells. The LHPP protein levels in the overexpression groups (1.1152 ± 0.0151) were significantly higher than those in the control groups (0.5240 ± 0.0469), the LHPP protein levels in the knockdown groups (0.2687 ± 0.0198) were significantly lower than those in the control groups (0.6055 ± 0.0475, *P* <0.001). (**C**) The overexpression and knockdown efficiencies were confirmed by quantitative PCR (**P <0.01,***P <0.001). (**D**) A periodical test by CCK-8 revealed that LHPP group cell lines exhibited lower viability and proliferation rate after 96 h, whereas the shLHPP group exhibited an increased level of cell viability (*P <0.05, **P <0.01,***P <0.001). (**E**) Transwell analysis for migration and invasion. The number of cells that passed through the membranes were counted using Crystal Violet-stained images (*P <0.05, **P <0.01). (**F**) Clone formation analysis showed that cell proliferation was significantly decreased in the LHPP group and significantly increased in the shLHPP group.

We confirmed the transfection efficiency using fluorescence (Figure 2A) and verified the overexpression and knockout efficiency at mRNA and protein levels (Figure 2B,C). Compared with the control group, the LHPP group showed decreased cell viability; however, the shLHPP exhibited increased cell viability (Figure 2D). Transwell analysis showed that migration and invasion of BxPC-3 and AsPC-1 cells were inhibited in the LHPP group, whereas the shLHPP group presented the opposite results (Figure 2E). Clone formation analysis showed that cell proliferation was significantly decreased in the LHPP group and increased in the shLHPP group (Figure 2F). These results suggested that LHPP suppressed the proliferation, migration, and invasion of PaCa cells.

### Study on the inhibitory mechanism of LHPP on proliferation, migration, and invasion of pancreatic cancer cells

**Figure 3:**
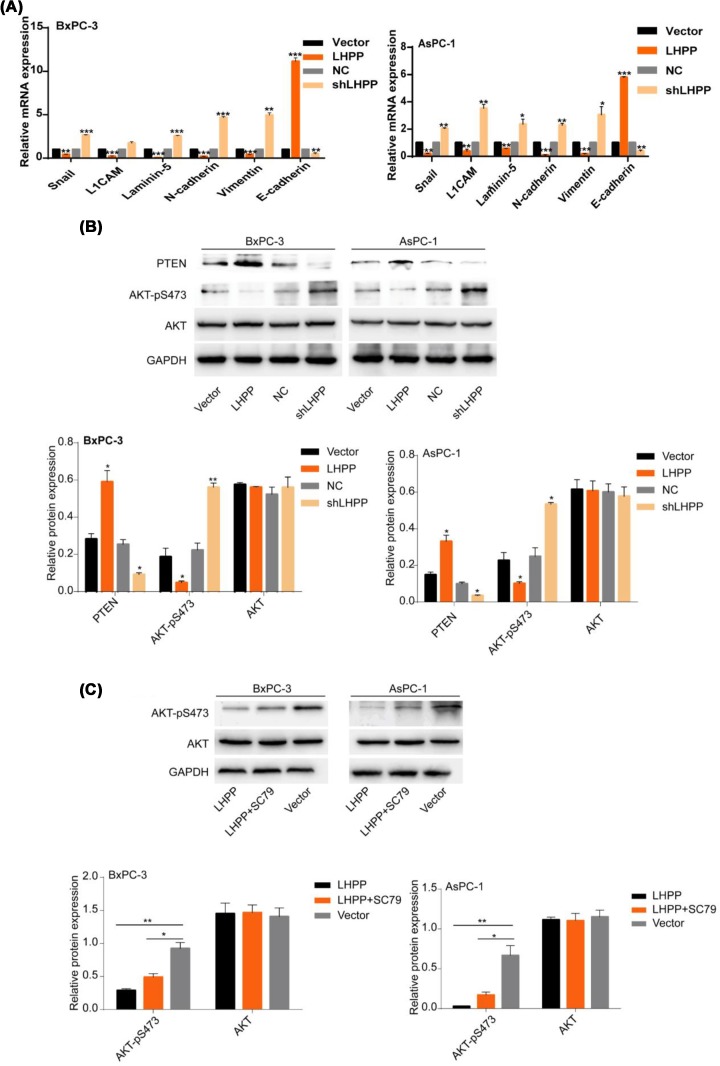
LHPP affects the expression of PTEN and AKT in pancreatic carcinoma (**A**) RT-qPCR analysis of snail, L1CAM, laminin-5, fibronectin, N-cadherin, vimentin and E-cadherin in the LHPP and shLHPP groups. (**B**) The expression levels of PTEN, p-AKT, and AKT were analyzed by western blot. (**C**) The LHPP group cells were pretreated with SC79; then, the correlation between p-AKT and the LHPP group was observed using western blot (*P <0.05, **P < 0.01, ***P <0.001).

The LHPP group showed significantly inhibited proliferation and transfer of PaCa cells, as evidenced by the reduced mRNA expression levels of snail, laminin-5, L1CAM, N-cadherin, and vimentin; the shLHPP group showed the opposite results (Figure 3A). As shown in Figure 3B, the LHPP group could promote PTEN expression and inhibit phosphorylated AKT expression, whereas the opposite was observed in the shLHPP group. The addition of the drug SC79 (an important AKT activator) to the LHPP group led to an increase in phosphorylated AKT (Figure 3C). In this experiment, we found that LHPP significantly reduced AKT phosphorylation and enhanced the expression of PTEN protein.

### Apoptosis of pancreatic cancer cells was induced by LHPP

**Figure 4:**
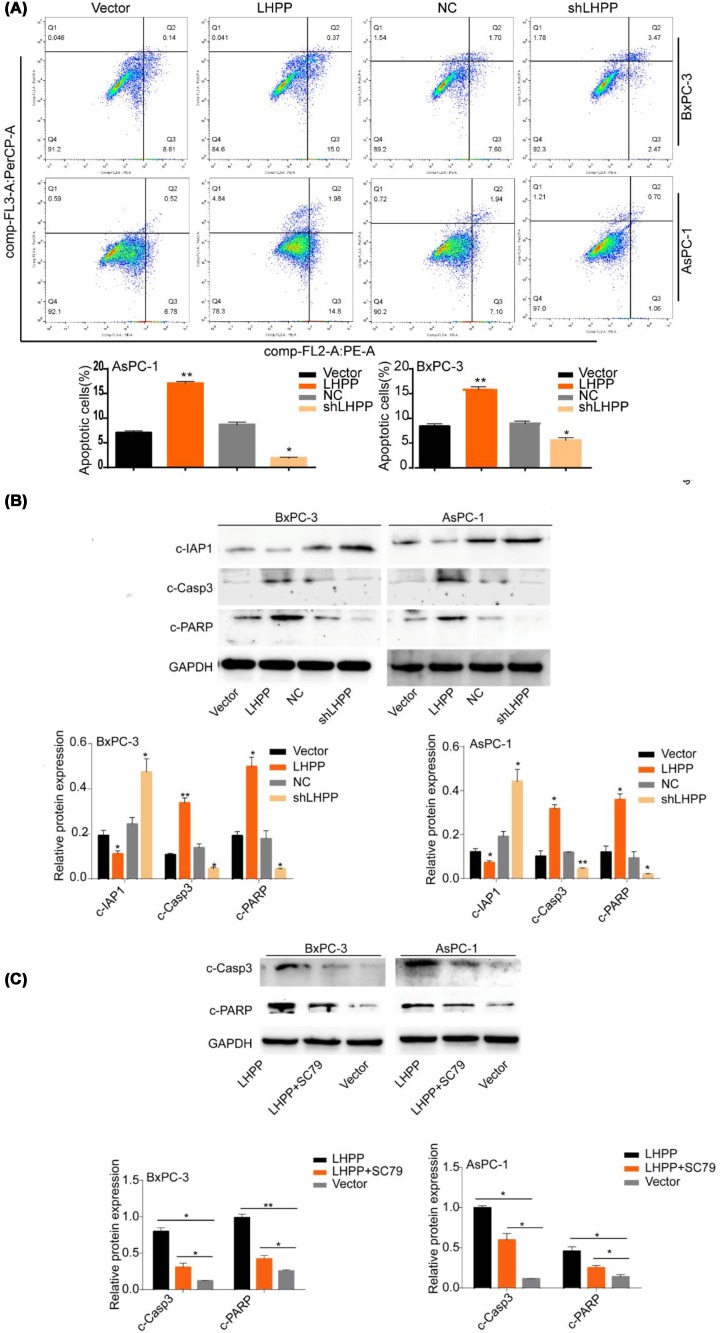
Apoptosis of pancreatic cancer cells was induced by LHPP (**A**) PaCa cell groups were subjected to flow cytometry to analyze apoptosis. Apoptosis was clearly observed in the LHPP group, but a lower ratio of apoptotic cells was observed in the shLHPP group. (**B**) The expression levels of cIAP1, cleaved-Casp3, and cleaved-PARP were analyzed by Western blot. (**C**) The PaCa cells were pretreated with SC79; then, the correlation between cleaved-Casp3 and cleaved-PARP and the LHPP group was observed using Western blot (*P <0.05, **P <0.01).

The passaging results showed that the apoptosis of BxPC-3 and AsPC-1 cells was promoted in the LHPP group, whereas the apoptosis rate was lower in the shLHPP group than that in the NC group (Figure 4A). Cleaved-PARP and cleaved-Casp3 are important proteins in the process of apoptosis, and we further found that the LHPP group showed promoted cIAP1 expression and inhibited levels of cleaved-PARP and cleaved-Casp3 in BxPC-3 and AsPC-1 cells; however, these results differed in the shLHPP group (Figure 4B). SC79 pretreatment in the LHPP group reduced the levels of cleaved-PARP and cleaved-Casp3 in PaCa cells (Figure 4C). These results suggested that LHPP could inhibit the apoptosis of PaCa cells.

## Discussion

It is important to explore the relevant mechanism to overcome the difficulties of PaCa treatment. LHPP is one of the few known mammalian histidine phosphatases and can dephosphorylate His-phosphorylated protein substrates *in vitro*, including succinate thiokinase and nucleoside diphosphate kinase [14]. LHPP plays an important role in cell signal transduction and metabolism. Furthermore, the histidine phosphatase LHPP has been found to be associated with cancer. Here, we found that LHPP expression decreased in PaCa tissues and cells, indicating that LHPP was a potential tumor suppressor, which is consistent with the findings in melanoma and liver, cervical, and bladder cancers [9–12]. In our experiment, we also found that the degree of PaCa differentiation was negatively correlated with LHPP expression, and LHPP expression in PaCa with lymph node metastasis was significantly lower than in that without lymph node metastasis. In addition, LHPP expression was significantly lower in HPDE6-C7 cells than that in pancreatic cancer cell lines. The LHPP group showed inhibited proliferation, migration and invasion and promoted apoptosis in PaCa cells. More importantly, we found that LHPP could regulate the PTEN/AKT signaling pathway in PaCa cells, and this pathway plays a key role in the migration, invasion, proliferation, and apoptosis of tumors.

Epithelial–mesenchymal transformation (EMT) induced by the transcription factor Snail is considered to be closely related to tumor invasion and metastasis [15,16]. Laminin-5 and L1CAM promote the highly malignant phenotype of PaCa cells [17,18]. The up-regulation of N-cadherin and vimentin is considered to be an indicator of the EMT process, which in turn is positively correlated with tumor invasion [19]. Our data showed that snail, L1CAM, laminin-5, N-cadherin, and vimentin were inhibited in the LHPP group. Therefore, our studies indicated that LHPP inhibited proliferation, migration, and invasion in PaCa cells.

PTEN can inhibit tumor progression, as it converts phosphatidylinositol-3,4,5-trisphosphate (PIP3) to phosphatidylinositol-4,5-bisphosphate (PIP2) at the cellular membrane; this causes inhibition of PI3K-AKT signaling, hence restraining tumor cell proliferation, migration, and invasion and promoting apoptosis [20,21]. As a downstream signal molecule of the PI3K signal transduction pathway, phosphorylated AKT has been found to be continuously highly activated in many kinds of tumors [22,23]. Our data showed that the LHPP group had enhanced PTEN expression and a decreased phosphorylated AKT protein level, whereas the shLHPP group showed the opposite results. Therefore, LHPP may regulate PaCa cell progression via the AKT signaling pathway, which plays an important role in the proliferation, migration, invasion, and apoptosis of cancer cells. We found that the addition of SC79 to the LHPP group promoted phosphorylated AKT expression, indicating that SC79 enhanced progression in PaCa cells by down-regulating LHPP. The inhibitory effect of LHPP on the development of pancreatic cancer cells may provide a new theoretical basis for the treatment of PaCa.

The process of apoptosis in cancer cells is mediated by the activation of caspases [24], and caspase-3 is an important promoter in apoptosis [25]. The activation of caspase-3 affects major structural proteins and activates other enzymes, leading to apoptosis. The cIAP1 gene supports tumor growth by restraining apoptosis; this anti-apoptotic activity is achieved by inhibiting specific caspases, and the proteolysis of caspase-3 is prevented by inhibiting cytochrome *c*-induced activation of caspase-9 [26,27]. Cleaved-PARP is the cleavage substrate of caspase, which is the core player in apoptosis [28]. Importantly, the AKT signaling pathway could inhibit the activity of cleaved-Casp3 [29]. We found that the LHPP group showed increased protein expression of cleaved-Casp3 and cleaved-PARP and decreased cIAP1 protein expression in PaCa cells, whereas the opposite results were observed in the shLHPP group. The results of flow cytometry showed that the LHPP group could significantly promote PaCa cell apoptosis, and the apoptosis rate in the shLHPP group was lower than that in the NC group. Therefore, LHPP could promote the apoptosis of PaCa cells. Pretreatment with SC79 in the LHPP group demonstrated that SC79 decreased the effect of LHPP on PaCa cell apoptosis. We assumed that LHPP could promote apoptosis through the AKT signaling pathway. In this experiment, we discovered that LHPP influenced PaCa cell progression by promoting apoptosis.

Several limitations in the present study should be acknowledged, First of all, sequencing technology and professional institutions should be carried out to distinguish the differences between BxPC-3 and AsPC-1 cell lines; second, due to the lack of research on LHPP at this stage, the specific mechanism is not clear, so there is no further study on the specific role of LHPP in AKT pathway, and the research is relatively superficial; finally, the present study only carried out experiments on BxPC-3 and AsPC-1 cells, and not in other PaCa cell lines.

In conclusion, the results suggested that LHPP had low expression in PaCa tissues and cell lines. LHPP could inhibit proliferation, migration, and invasion and enhance apoptosis through the AKT signaling pathway in PaCa cells. Therefore, LHPP may provide a new strategy for the treatment of PaCa cells in the future.

## Abbreviations

Casp-3: cysteinyl aspartate specific proteinase
CCK-8: Cell Counting Kit-8
cIAP1: cellular inhibitor of apoptosis protein
EMT: epithelial–mesenchymal transformation
MOD: Mean Optical Density
PaCa: pancreatic cancer
PARP: poly ADP-ribose polymerase
PIP2: phosphatidylinositol-4,5-bisphosphate
PIP3: phosphatidylinositol-3,4,5-trisphosphate
PTEN: Phosphatase and tensin homolog
PVDF: polyvinylidenefluoride membranes
